# Quantile-Specific Penetrance of Genes Affecting Lipoproteins, Adiposity and Height

**DOI:** 10.1371/journal.pone.0028764

**Published:** 2012-01-03

**Authors:** Paul T. Williams

**Affiliations:** Lawrence Berkeley National Laboratory, Berkeley, California, United States of America; University of Tor Vergata, Italy

## Abstract

Q*uantile-dependent penetrance* is proposed to occur when the phenotypic expression of a SNP depends upon the population percentile of the phenotype. To illustrate the phenomenon, quantiles of height, body mass index (BMI), and plasma lipids and lipoproteins were compared to genetic risk scores (GRS) derived from single nucleotide polymorphisms (SNP)s having established genome-wide significance: 180 SNPs for height, 32 for BMI, 37 for low-density lipoprotein (LDL)-cholesterol, 47 for high-density lipoprotein (HDL)-cholesterol, 52 for total cholesterol, and 31 for triglycerides in 1930 subjects. Both phenotypes and GRSs were adjusted for sex, age, study, and smoking status. Quantile regression showed that the slope of the genotype-phenotype relationships increased with the percentile of BMI (P = 0.002), LDL-cholesterol (P = 3×10^−8^), HDL-cholesterol (P = 5×10^−6^), total cholesterol (P = 2.5×10^−6^), and triglyceride distribution (P = 7.5×10^−6^), but not height (P = 0.09). Compared to a GRS's phenotypic effect at the 10^th^ population percentile, its effect at the 90^th^ percentile was 4.2-fold greater for BMI, 4.9-fold greater for LDL-cholesterol, 1.9-fold greater for HDL-cholesterol, 3.1-fold greater for total cholesterol, and 3.3-fold greater for triglycerides. Moreover, the effect of the rs1558902 (FTO) risk allele was 6.7-fold greater at the 90^th^ than the 10^th^ percentile of the BMI distribution, and that of the rs3764261 (CETP) risk allele was 2.4-fold greater at the 90^th^ than the 10^th^ percentile of the HDL-cholesterol distribution. Conceptually, it maybe useful to distinguish environmental effects on the phenotype that in turn alters a gene's phenotypic expression (quantile-dependent penetrance) from environmental effects affecting the gene's phenotypic expression directly (gene-environment interaction).

## Introduction

Genome-wide association studies have shown that for most traits, a few, common, single nucleotide polymorphisms (SNP) account for a small proportion of the genetic variance [1]. Meta-analyses have been instrumental in culling a select subset of true associations from the large number of false positive results [2]. With respect to the analyses of the data per se, statistical concerns have focused on adjustment for covariates, transformations for nonnormal phenotypes, and selection of additive vs. dominant phenotypic expression of the allelic dose [3]. Major questions remain as to why SNPs explain only small proportions of the phenotypic variance for traits showing high genetic inheritance from twin and family studies [4].

The classical regression model assumes that the relationship between the independent variable (e.g., genotype) and dependent variable (e.g., phenotype) applies to all quantiles of the dependent variable [5]. For example, the 0.39 kg/m^2^ per allele increase in body mass index (BMI) for the rs1558902 (FTO gene) [6] is assumed to apply equally to healthy weight, overweight, and obese individuals. There is, however, no a priori biological rationale for this premise.

We hypothesize that describing the effect of single nucleotide polymorphisms (SNP) by their standard regression slope may fundamentally mischaracterize their relationship, and contribute in a modest way to underestimating the proportion of the variance explained by genetic variants. Specifically, some of the missing genetic variance could be due to the misperception that the same genotype-phenotype relationship applies whether the phenotypic value is high, intermediate, or low relative to its population distribution. Although there are often statistical advantages to comparing the genotypic frequencies at the phenotypic extremes [7], differing penetrance for the tails of the distribution would also argue against comparing their genotypic frequencies to identify their genetic determinants.

To test this hypothesis, this paper examines the relationships of linear combinations of SNPs shown to predict lipoprotein concentrations, BMI, and height in published meta-analyses [6], [8], [9]. Genetic risk scores (GRS) for BMI, plasma lipid and lipoprotein concentrations, and height were created from the published meta-analyses of individuals of European ancestry (Table 1 in Speliotes et al., 2010 [6]; supplementary Table 2 in Teslovich et al., 2010 [8], supplementary Table 1 in Lango et al., 2010 [9]). The meta-analyses identified genome-wide statistical significance for 32 SNPs with BMI, 37 SNPs with low-density lipoprotein (LDL)-cholesterol concentrations, 47 SNPs with high-density lipoprotein (HDL)-cholesterol concentrations, 52 SNPs with total cholesterol concentrations, 31 SNPs with plasma triglyceride concentrations, and 180 loci for height. Each individual was given a GRS that was the summation of the product of the number of minor alleles for each SNP and their published per allele phenotypic effect (e.g., #minor alleles SNP1*its published per allele effect on the phenotype + #minor alleles SNP2*its published per allele effect on the phenotype,….). Height, BMI, lipids, and GRSs were adjusted for sex, age (age and age^2^), study, and smoking status. In each case, the unit of measure of the GRS was the predicted kg/m^2^ (BMI), mg/dL (lipids and lipoproteins), or z-score increase (height). In addition, two SNPs are examined that have shown consistent replication across multiple studies: BMI vs. rs1558902 (FTO) [6], and HDL-cholesterol vs. rs3764261 (CETP) [8]. The results suggest that phenotypic expressions of SNPs are significantly related to the percentile of the lipoprotein and BMI distribution, and that measuring a SNP's effect by the standard regression slope may underestimate its true genetic impact. The consistency of the results across multiple SNPs and traits suggest this phenomenon may not be uncommon.

## Results

### LDL-cholesterol

**Figure 1:**
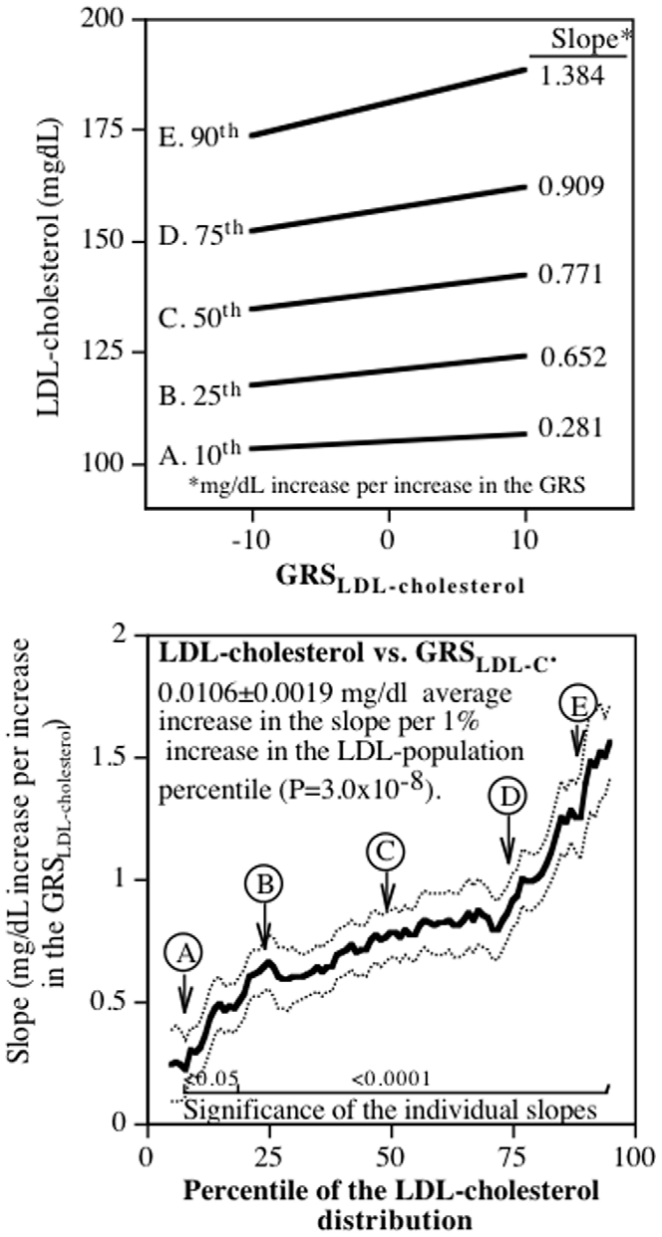
Increase in LDL-cholesterol per increase in the GRS_LDL-cholesterol_ for selected percentiles (upper panel), and for all percentiles as a function of the LDL-percent distribution (lower panel). Note that the Y-axis represents LDL-cholesterol concentrations in the upper panel, and the slopes for LDL-cholesterol vs. GRS_LDL-cholesterol_ in the lower panel. The correspondence between the upper and lower panels is illustrated by the letter designation of the corresponding slopes at the 10^th^ (A), 25^th^ (B), 50^th^ (C), 75^th^ (D), and 90^th^ (E) LDL-percentile distribution. Lighter lines designate ± one standard error.

Standard regression analyses showed that when adjusted for covariates, plasma LDL-cholesterol concentrations increased (slope±SE) 0.801±0.085 mg/dL per unit increase in the GRS_LDL-cholesterol_ (Table 1, 4.4% of the variance, P<10^−15^). Figure 1 (upper panel) presents the regression analyses for selected quantiles of the LDL-cholesterol distribution. It shows that the slopes became progressively larger at the higher quantiles of the LDL-distribution. These slopes, along with the slopes for the other quantiles, are presented in the lower panel's graph of the regression slopes (Y-axis) as a function of the quantile of the LDL-cholesterol distribution (X-axis). The Y-axis of the lower panel represents the slopes rather than the LDL-cholesterol concentrations themselves (compare with the upper panel). Specifically, the Y-axis represents the LDL-cholesterol vs. GRS_LDL-cholesterol_ slope at the 5^th^ quantile of the LDL-cholesterol distribution, the 6^th^ quantile of the LDL-cholesterol distribution,…, and the 95^th^ quantiles of the LDL-cholesterol distribution. Dashed lines present the standard errors for the slopes at each quantile value. The figure shows that each unit increase in the GRS_LDL-cholesterol_ was associated with an LDL-cholesterol increase of 0.281±0.099 mg/dL at the 10^th^ percentile of the LDL-distribution, 0.652±0.111 mg/dL at the 25^th^ percentile, 0.771±0.098 mg/dL at the 50^th^ percentile (the median), 0.909±0.110 mg/dL at the 75^th^ percentile, and 1.384±0.195 mg/dL at the 90^th^ percentile of the LDL-cholesterol distribution. If the slopes relating LDL-cholesterol to the GRS_LDL-cholesterol_ were the same throughout the LDL-cholesterol distribution, as traditionally assumed, then the upper graph would present parallel regression lines, and the lower graph would present a simple horizontal line. In fact, the graph shows that the increase in LDL-cholesterol became progressively more positive with increasing percentile of the its distribution, such that on average each 1-percent increase was associated with a 0.0106±0.0019 mg/dL increase in the slope (P = 3×10^−8^). The LDL-cholesterol-GRS slope was 4.93-fold greater at the 90^th^ than at the 10^th^ LDL-cholesterol percentile.

**Table 1:** Standard least-squares and quantile regression analyses of lipids, lipoproteins, BMI and height.

	Quantile regression (slope±SE)					
	10^th^ percentile	25^th^ percentile	50^th^ percentile	75^th^ percentile	90^th^ percentile	Standard least squares regression
LDL-cholesterol (mg/dL) vs. GRS	0.28±0.10	0.65±0.11	0.77±0.10	0.91±0.11	1.38±0.20	0.80±0.09
HDL-cholesterol (mg/dL) vs. GRS	0.69±0.13	0.69±0.10	0.78±0.08	0.98±0.10	1.29±0.15	0.86±0.07
HDL-cholesterol (mg/dL) vs. CETP (rs3764261)	2.27±0.60	2.69±0.43	2.96±0.44	4.38±0.67	5.51±1.01	3.37±0.40
Total cholesterol (mg/dL) vs. GRS	0.43±0.10	0.56±0.09	0.70±0.08	1.07±0.12	1.32±0.18	1.01±0.09
Triglycerides (mg/dL) vs. GRS	0.74±0.14	0.79±0.11	1.16±0.13	1.59±0.29	2.46±0.42	1.36±0.17
BMI (kg/m^2^) vs. GRS	0.44±0.27	0.36±0.19	1.19±0.26	1.52±0.30	1.88±0.59	0.98±0.23
BMI (kg/m^2^) vs. FTO (rs1558902)	0.22±0.24	0.09±0.19	0.62±0.26	1.07±0.21	1.47±0.45	0.82±0.20
Height (z-score) vs. GRS	1.12±0.16	1.18±0.13	1.17±0.09	0.98±0.08	0.93±0.13	1.09±0.08

The absolute difference in slopes at the 10^th^ and 90^th^ quantiles (1.10 mg/dL) exceeded the traditional regression slope (0.801 mg/dL) by 38%. The 95% confidence interval for the standard regression slope (i.e., ±1.96*SE) included only those slopes between the 24^th^ and 77^th^ quantiles of Figure 1, misrepresenting the LDL-cholesterol-GRS slope for 46% of the LDL-cholesterol distribution. Allowing the slopes to increase with the quantiles of the LDL-distribution improved the proportion of the variance explained by the GRS_LDL-cholesterol_ by 20.1% (from 4.43% to 5.61% of the variance).

### HDL-cholesterol

**Figure 2 pone-0028764-g002:**
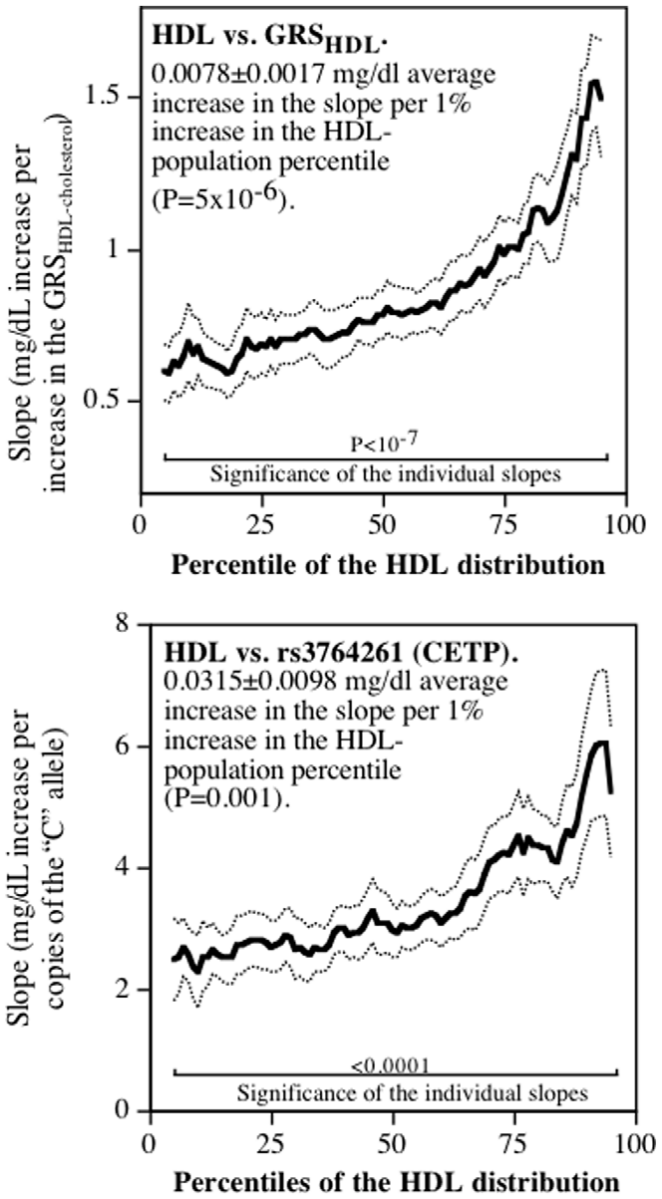
Slopes for HDL-cholesterol versus GRS_HDL-cholesterol_ and the number of C alleles for rs3764261 (CEPT, Y-axis) by percentiles of the HDL-cholesterol distribution (X-axis). Lighter lines designate ± one standard error.

Standard regression analyses showed that when adjusted for covariates, plasma HDL-cholesterol concentrations increased (slope±SE) 0.860±0.074 mg/dL per increase in the GRS_HDL-cholesterol_ (6.6% of the variance, P<10^−15^), of which more than one-half can be ascribed to the number of C alleles of rs3764261 (slope±SE: 3.369±0.398 mg/dl per dose of the risk allele, explaining 3.58% of the variance, P<10^−15^). Figure 2 shows that the effects of both GRS_HDL-cholesterol_ and rs3764261 increased in proportion to the quantiles of the HDL distribution (P<0.0001). Compared to their slope at the 10^th^ percentile, the slope at the 90^th^ HDL percentile was 1.87- and 2.42-fold greater for GRS_HDL-cholesterol_ and rs3764261, respectively. The 95% confidence interval for the standard regression slopes included only between the 35^th^ and 76^th^ percentile of the HDL cholesterol distribution for the GRS_HDL-cholesterol_ (misrepresenting 58%), and excluded those slopes above the 65^th^ HDL percentile for rs3764261. The absolute differences in the slopes between the 10^th^ and 90^th^ percentiles represented 70% of the standard regression estimate for the GRS_HDL-cholesterol_ score, and 96% for rs3764261. We estimate that allowing the regression slope to increase with the percentile of the lipoprotein distribution increased the percent of the variance explained by 7.6% for the GRS_HDL-cholesterol_ (from 6.61 to 7.11), and by 6.1% for rs3764261 (from 3.58% to 3.80%).

### Lipids

**Figure 3 pone-0028764-g003:**
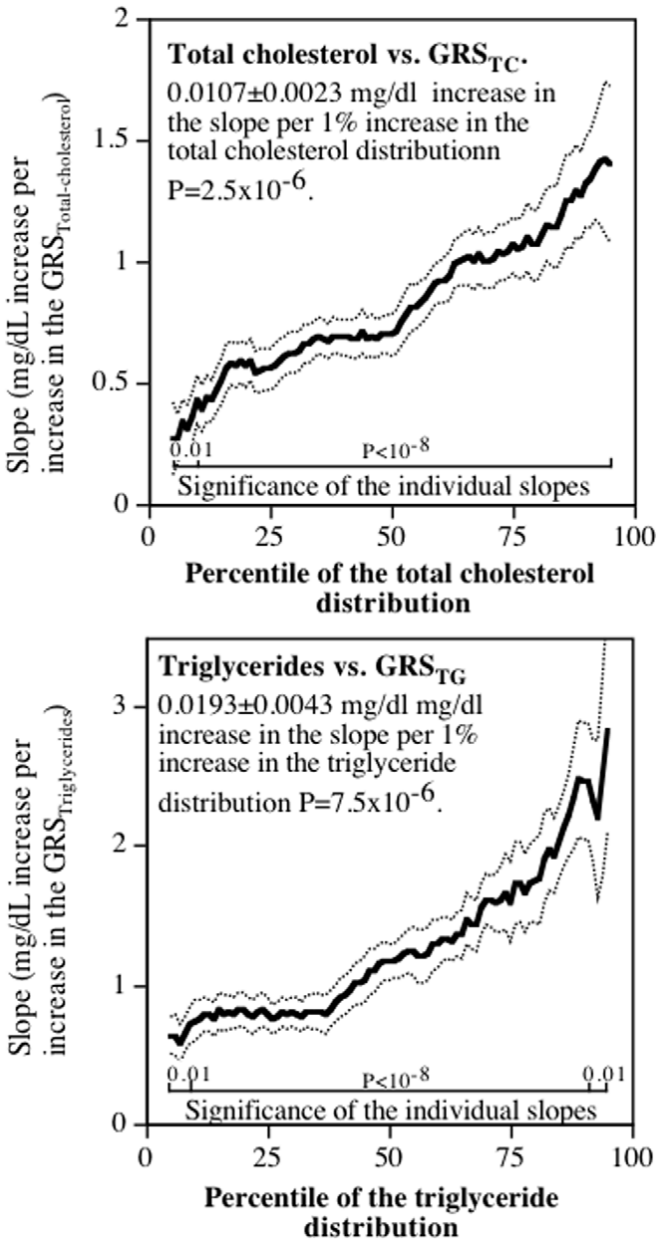
Slopes for plasma total cholesterol concentrations versus GRS_Total cholesterol_ and plasma triglyceride concentrations versus GRS_Triglycerides_ (Y-axis) by the percentiles of the lipid distribution (X-axis). Lighter lines designate ± one standard error.

Standard regression analyses showed that when adjusted for covariates, plasma total cholesterol concentrations increased (slope±SE) 1.011±0.088 mg/dL per increase in its GRS (6.4% of the variance, P<10^−15^), and plasma triglycerides increased 1.359±0.167 mg/dL per increase in its GRS (3.3% of the variance, P<10^−15^). Figure 3 showed that their slopes with GRS_Total cholesterol_ and GRS_Triglycerides_ increased significantly with increasing quantiles of their distributions. Whereas total cholesterol showed a mostly linear increase (acceleration) with increasing quantile values, the graph for plasma triglyceride concentrations suggested a steeper rise in its regression slopes with increasing percentiles of the triglyceride distribution. Compared to their slopes at the 10^th^ percentile, the increase in slope was 3.07-fold larger for the 90^th^ percentile of the total cholesterol distribution, and 3.34-fold larger for the 90^th^ percentile of plasma triglycerides. The 95% confidence interval for the standard regression slopes included only between the 57th and 85th percentiles of the total cholesterol distribution (misrepresenting 71%), and between the 46th and 76th percentiles of the triglyceride distribution (misrepresenting 69%). The absolute differences in the slopes between the 10th and 90th percentiles exceeded the standard regression estimate by 27% for triglycerides, and represented about 88% of the standard regression estimate for total cholesterol. We estimate that allowing the regression slope to increase with the percentile of the lipoprotein distribution increased the percent of the variance explained by 4.6% for total cholesterol (from to 6.40% to 6.70%) and by 27.9% for plasma triglyceride concentrations (from 3.31% to 4.23%).

### Body mass index

Standard regression analyses showed that when adjusted for covariates, BMI increased (slope±SE) 0.982±0.227 kg/m^2^ per increase in the GRS_BMI_ (0.96% of the variance, P = 1.6×10^−5^), almost all of which was explained by the number of T alleles for rs1558902 in the FTO gene (slope±SE: 0.815±201 kg/m^2^ per dose of the risk allele, P = 5×10^−5^, 0.85% of the variance explained). The proportion of the BMI variance explained was improved by fitting separate coefficients to rs1558902 and to the weighted combination of the 31 other SNPs (total percent of the variance explained: 1.15%). The GRS_BMI_ was therefore defined by 0.807904 rs1558902+ 0.708466*the weighted combination of the 31 other SNPs, which predicted a 1.000±0.210 kg/m^2^ increase per increase in the GRS_BMI_ (P = 1.8×10^−6^).

**Figure 4:**
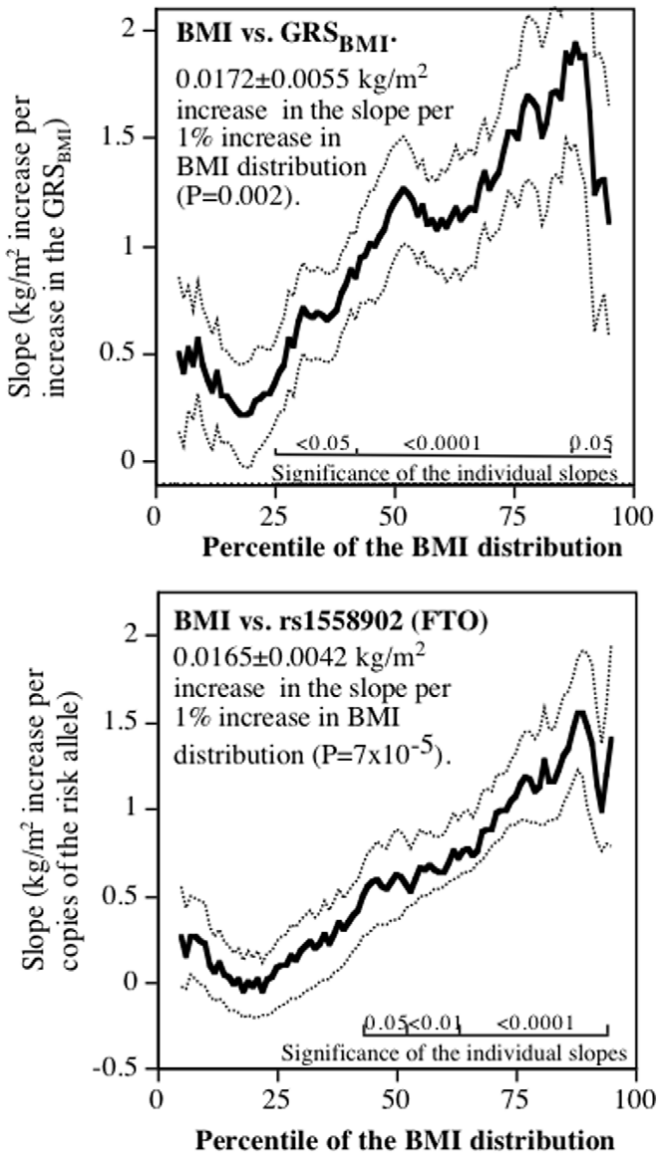
Slopes for BMI versus GRS_BMI_ and rs155890 (FTO gene, Y-axis) by the percentile of the BMI distribution (X-axis). Lighter lines designate ± one standard error.

Figure 4 displays the plot of the regression slopes for both the GRS_BMI_ and rs1558902 by the quantiles of the BMI distribution. For the GRS_BMI_, the regression slope increased 0.01721±0.0055 kg/m^2^ for each 1% increment in the BMI percentile (P<0.0001). For rs1558902, the regression slope increased 0.0165±0.0042 kg/m^2^ for each 1% increment in the BMI percentile (P<0.0001). The GRS_BMI_ had a 4.24-fold greater effect, and rs1558902 had 6.69-fold greater effect, at the 90^th^ percentile than at the 10^th^ BMI percentile. The figures display generally linear increases in the slope with inflections at the extremes where the precision in estimate diminishes. The 95% confidence interval for the standard regression slope included only those slopes between the 29^th^ and the 73^rd^ BMI percentiles for GRS_BMI_, and between the 42^rd^ and 75^th^ BMI percentiles for rs1558902. Allowing the regression slope to increase with the percentile of the BMI distribution increased the percent of the variance explained by 24.7% for GRS_BMI_ (from 1.15 to 1.43) and by 59.1% for rs1558902 (from 0.79 to 1.26).

### Height

**Figure 5:**
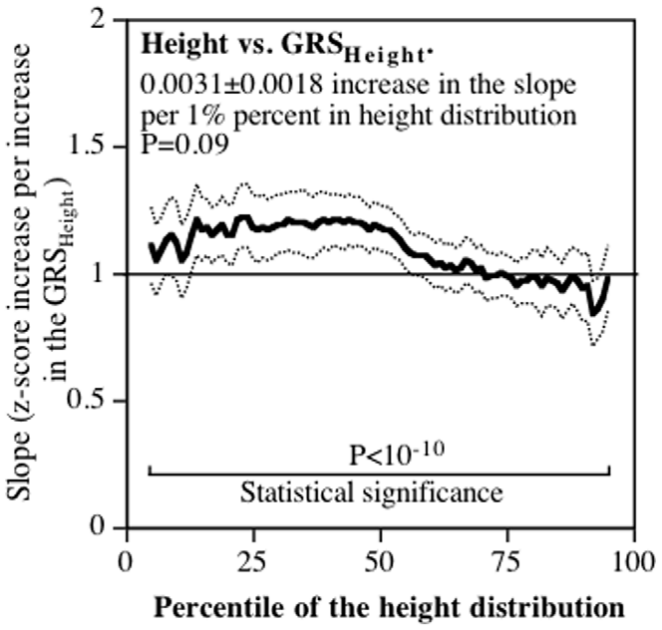
Slopes for height versus GRS_Height_ (Y-axis) by the percentile of the height distribution (X-axis). Lighter lines designate ± one standard error.

Standard regression analyses showed that when adjusted for covariates, the z-score for height increased 1.086±0.076 units per increase in the GRS_Height_ (9.66% of the variance, P<10^−15^). Figure 5 shows that the regression slope did not increase with the percentile of the height distribution. With minor exceptions (86^th^, 92^nd^–94^th^ percentiles), the 95% confidence interval for the standard regression slope included all slopes for height vs. GRS_Height_.

### Additional analyses

The preceding analyses were redone using weights for the individual SNPs that maximize the proportion of the variance explained in the current sample, rather than the published effects from meta-analyses. Using multiple regression to find the best weights for our specific sample increased the explained variance from 4.4% to 7.2% for LDL-cholesterol, from 6.6% to 8.8% for HDL-cholesterol, from 6.4% to 9.5% for total cholesterol, from 3.3% to 4.4% for triglycerides, and from 1.15% to 3.09% for BMI. Adjusting for the number of coefficients fitted had little effect on the percent of the variance explained (i.e., adjusted R^2^). On average, each 1% increase in the quantile of the dependent variable was associated with a 0.0106±0.0022 increase in the slope for LDL-cholesterol vs. GRS_LDL-cholesterol fitted_ (P<10^−6^), a 0.0102±0.0017 mg/dL increase in the slope for HDL-cholesterol vs. GRS_HDL-cholesterol fitted_ (P = 2.4×10^−9^), a 0.0069±0.0018 mg/dL increase in the slope for total cholesterol vs. GRS_Total cholesterol fitted_ (P = 5×10^−5^) a 0.0142±0.0027 mg/dL increase in the slope for triglycerides vs. GRS_triglycerides fitted_ (P = 10^−7^), and a 0.0145±0.0032 kg/m^2^ increase in the slope for BMI vs. GRS_BMI fitted_ (P = 10^−6^). Compared to the 10^th^ quantile of the dependent variable, the regression slope at the 90^th^ quantile was 2.93-fold larger for LDL-cholesterol vs. GRS_LDL-cholesterol fitted_, 2.23-fold larger for HDL-cholesterol vs. GRS_HDL-cholesterol fitted_, 1.78-fold larger for total cholesterol vs. GRS_total cholesterol fitted_, 3.26-fold larger for triglycerides vs. GRS_triglycerides fitted_, and 3.73-fold larger for BMI vs. GRS_BMI fitted_. We estimate that allowing the regression slopes to increase with the percentile of the independent variable increased the percent of the variance explained by 15.6% for LDL-cholesterol (from 7.19 to 8.31), 12.5% for HDL-cholesterol (from 8.82 to 9.92), 6.59% for total cholesterol (from 9.48 to 10.11), 42.1% for triglycerides (from 4.40 to 6.25), and 20.1% for BMI (from 3.09 to 3.71). Thus the increases in the regression slopes with the percentiles of the dependent variable persisted. We also verified that the reported findings were not artifacts of skewness or other distributional characteristics of the dependent variable. Specifically, randomly permuting the residuals across the fitted standard regression estimates, thereby insuring parallel increases for all quantiles of the dependent variable, produced no significant relationship between the regression slopes and the quantiles of the dependent variable (analyses not displayed).

## Discussion

We have shown that across a variety of traits the phenotypic expression of genetic variation differed by the percentile of the phenotype. We are aware of no reference to this phenomenon in the various reviews of the analyses of SNPs. Forsooth, if the effects of SNPs on the phenotypes as estimated by standard regression analyses merit scientific significance, then so must their differences across the percentile distribution of the trait, being nearly as great or greater than the standard regression estimates themselves. The phenomenon was demonstrated for both GRS calculated from the published effects of allelic dose and GRS calculated from individual effects that maximize the percent of the variance explained for our specific dataset. These analyses do not reveal whether these genotypes are specifically responsible for the more extreme phenotype values, or whether the penetrance of these genotypes was greater in subjects in the higher percentiles of the population. Figures 1, 2, 3, 4 all show that the phenotypic variance increases with the GRS, which would likely affect variance-component estimates in genetic models, particularly in cases where the significance of the effects are marginal. We believe the phenomenon is common, and has key implications with respect to estimating the proportion of the variance explained, the study of gene-environment interactions, and the design of studies.

### Ubiquity

Genotypic expression was shown to depend upon the percentile of the phenotype distribution for GRSs representing the combined effects of 31 to 52 loci. Although a few SNPs had large effects, the majority of the GRSs represented the sum of a large number of small to moderate size effects. Averaging over different genotype-phenotype relationships within each GRS, some increasing, some decreasing, and some showing no difference across the phenotype's quantile distribution, might be expected to cancel each other out, converging to the classical statistical model of the same slope throughout the phenotype distribution, but this was not observed. Although we lacked the statistical power to assess this phenomenon for individual SNPs, their collective effect in the GRS suggests that the majority of their individual effect must also be quantile dependent. Moreover, we demonstrated that the phenotypic expressions of the two SNPs with the strongest association with their trait (i.e., rs3764261 vs. HDL-cholesterol, and rs1558902 vs. BMI) increased significantly with the percentile of the trait distribution.

### Proportion of the variance explained

Considerable effort and expense has been spent on identifying the associations between SNPs and traits that individually usually explain very small portions of the phenotypic variance [1], [4]. However, the combined influences of multiple SNPs into genetic risk scores have begun to approach the contribution of other risk factors. Standard regression analyses captured much of the phenotypic effect associated with the genotypes examined in this report. However, our analyses suggest that allowing the genotype's phenotypic expression to vary with the percentile of the trait distribution significantly increases the proportions of phenotypic variances explained. Allowing the rs1558902-BMI slope to increase by 1.8127*BMI_quantile_-0.27054 for each percent increase in the BMI distribution produced a larger increase in the percent of the BMI variance explained than did the addition of all 31 other SNPs currently associated with BMI at genome-wide statistical significance.

### Gene-environment interactions

**Figure 6:**
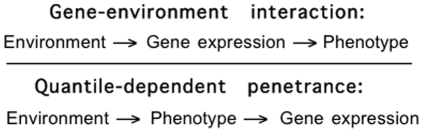
Suggested interpretation of quantile-dependent penetrance and gene-environment interaction.

Gene-environment interactions are surmised when the phenotypic expression of a genetic variant is altered by environmental status. It has been proposed that such interaction may contribute to the missing heritability [4]. However, if the phenotypic expression of a genotype is quantile dependent, then an environmental factor affecting the phenotype might increase or diminish the expression of the genotype. This would follow from the relationship of the genotype's effect to the percentile of the phenotype, rather than directly affecting the genotype's expression (Figure 6).

For example, the effect of rs155890 on BMI may be greater for individuals who are more obese (Figure 4). A number of recent articles have described a diminished effect of FTO polymorphisms on BMI in physically active versus sedentary individuals [10]–[13]. The effect is universally described as a gene-environment interaction [10]–[13]. However, Figure 4 suggests an alternative explanation based on the fact that the effect of the FTO polymorphism is diminished in lean vis-à-vis overweight individuals. Physically active individuals are leaner than sedentary individuals because exercise causes weight loss acutely and attenuates age-related weight gain in the long term [14], [15]. Thus, the apparent diminished effect of the FTO polymorphism with greater physical activity may be, in part, a direct consequence of the relationship of the genotype to quantiles of BMI as shown in figure 4.

Figure 2 shows that the effect of rs3764261 of the CETP gene on plasma HDL-cholesterol levels is quantile dependent. The Etude Cas-Temoin sur 1'Infarctus du Myocarde Study reported a strong interaction between CETP genotypes and alcohol on HDL-cholesterol, the genotype effect purported to be absent in teetotalers, and to increase progressively with increased alcohol consumption [16]. Similar interactions were cited for other HDL-related variables [16]. We hypothesize that this interaction could be due, in part, to quantile dependence causing the effect of CETP polymorphisms on HDL-cholesterol to be less for the lower HDL-cholesterol levels of the teetotalers and greater for higher HDL-cholesterol levels of heavy alcohol consumers. Similarly, reported associations between CETP polymorphisms and the HDL-cholesterol response to physical activity [17], [18] may also reflect, in part, quantile dependence in the relationships of CETP polymorphisms on plasma HDL-cholesterol concentrations.

### Experimental design

The best estimate of the standard regression slope is obtained by sampling data from the two ends of the range of the independent variable. This is part of the rationale for genetic studies that compare the upper tail of a trait's distribution, presumably enriched with high-risk genotypes, with the lower tail, presumably enriched with low-risk genotypes [7]. However, Figures 1 through [Fig pone-0028764-g002] [Fig pone-0028764-g003] 4 suggest that such comparisons may actually be between regions of the distribution having high genetic penetrance (upper tail) and low genetic penetrance (lower tail). The figures suggest that in some cases, a more informative design may be to restrict sampling to the upper population quantiles of a trait where phenotypic differences between high- and low-risk alleles are more fully expressed.

### Quantile dependence generally as a biological phenomenon

The effect of the percentile of the trait distribution on factors affecting BMI and lipoprotein concentrations is not limited to their genetic determinants. Elsewhere we have shown that the associations of moderate-intensity physical activity (i.e., walking) and vigorous-intensity physical activity (i.e., running) on BMI became progressively greater with increasing percentiles of the BMI distribution [19]–[22]. We have also reported that the well-established increase in HDL-cholesterol per unit alcohol intake was at least twice as great at the 95^th^ as at the 5^th^ quantile of the HDL distribution [23]. There was also a significant graded increase from the 5^th^ to the 95^th^ HDL percentile for the slopes relating HDL to exercise [23]. Men's HDL-cholesterol concentrations declined in association with fatness (BMI, waist, and chest circumference) more sharply at the 95^th^ than at the 5^th^ percentile of the HDL distribution [23]. BMI is a major determinant of plasma triglyceride levels, and we have shown that compared to the 5^th^ quantile of the triglyceride distribution, the rise in triglycerides at the 95^th^ quantile per unit of adiposity was 14-fold greater for BMI and 7.8-fold greater for waist circumference in men, and 8-fold greater for BMI in women [24]. The greater increases in triglycerides per unit of adiposity in whites than blacks, in men than women, and in LDL-pattern B compared to pattern A reported by others could all be explained, at least in part, to the dependence on the triglyceride population percentiles we reported [24].

### Nonnormality and data transformations

Quantile regression is a nonparametric technique and therefore there is no assumption of normality. Fitting the standard regression line to all the data and then randomly permuting the residuals among the fitted values did not produce the increases in the regression slope with the percentiles of the phenotype distributions, as would be expected (analyses not displayed). However, the functions in the figures could be used to transform the data such that the same relationship applies to all percentiles of the phenotype. In some cases, the transformation may approximate a log transformation, suggesting multiplicative rather than additive genetic effects.

### Conclusion

Our analyses suggest that the most important gene-environment interaction involves an individual's physiological environment within which genes are expressed. The lowest to the highest percentiles of a physiological trait represent range of physiologic parameters, genetic make-ups, and gene-gene interactions whose presence may be essential for the genetic variant to be expressed. The higher phenotypic range may represent a less regulated physiological environment than at lower phenotypic levels and providing more abundant substrates upon which the variant's expression depends. The relationships we observed for the GRSs are unlikely due to the effects of a few SNPs, since in most cases the effects of the individual SNPs are small. The greater genetic influence at higher physiological values may represent the influence of multiple loci, including enhanced synergism of gene-gene interaction.

## Materials and Methods

### Human subjects

This report uses the baseline data for Caucasian participants of the Cholesterol/Atherosclerosis Pharmacogenetics (CAP) Study and the Pravastatin Inflammation/CRP Evaluation (PRINCE) trial [25]–[28]. The characteristics of the samples have been previously published [25]–[27]. CAP subjects were recruited from two clinical centers: University of California, Los Angeles, School of Medicine (Los Angeles, CA) and San Francisco General Hospital (San Francisco, CA) [25]. PRINCE subjects were enrolled from 1143 sites representing 49 states and the District of Columbia, with no single site enrolling more than 4 patients [26], [27]. They were recruited on the basis of having serum total cholesterol levels of 4.14–10.36 mmol/L (CAP) or for having an LDL-cholesterol concentration ≥3.5 mmol/L or a history of myocardial infarction, stroke, or coronary revascularization regardless of their baseline LDL-cholesterol (PRINCE). Both studies excluded subjects for baseline use of statins or other lipid lowering agents, pregnancy, lactation, alcohol or drug abuse, liver disease, known statin intolerance, uncontrolled diabetes, uncontrolled thyroid disease or abnormal thyroid function, and likelihood for not completing the planned study based on the judgment of their physician (PRINCE) or <90% compliance with the study medication during a two-week run in period (CAP). The studies differed slightly with respect to minimum age (30 and 21 years old for CAP and PRINCE, respectively). The CAP study also excluded persons for serum triglycerides >4.52 mmol/L or fasting glucose >6.99 mmol/L; recent or planned change in diet or a weight change of ≥4.5 kg; the use of corticosteroids, immunosuppressive drugs, or drugs affecting the CYP3A4 system; elevated creatine phosphokinase levels >10 times the upper limits of normal; uncontrolled hypertriglyceridemia or blood pressure; abnormal renal function; or recent major illness in the preceding 3 months. Additional exclusion criteria for PRINCE were history of systemic inflammatory diseases (rheumatoid arthritis, osteoarthritis, inflammatory bowel disease, systemic lupus erythematous), myositis/myopathic process, or cancer; and use of steroids or chemotherapeutic drugs within the past year or chronic use of nonsteroidal anti-inflammatory drugs besides aspirin (use for ≥2 weeks within the past year). Human use approval was provided by the Lawrence Berkeley National Laboratory Human Subjects Committee and all participants signed statements of informed consent, which included permission for samples to be used in future genomic studies.

### Laboratory measurements

Plasma LDL-cholesterol concentrations in PRINCE were measured by a Centers for Disease Control and Prevention–standardized laboratory. For CAP, plasma total cholesterol and triglyceride concentrations were determined by enzymatic procedures on an Express 550 Plus analyzer (Ciba Corning, Oberlin, OH) and were consistently in control as monitored by the CDC-NHLBI standardization program. High-density lipoprotein (HDL)-cholesterol was measured after dextran sulfate precipitation of plasma [29], and LDL-cholesterol was calculated using the Friedewald formula [30]. Blood specimens from each subject were obtained after an overnight fast.

### Genotyping

Genotyping was performed in two stages: 1) 304 CAP and 675 PRINCE participants were genotyped for 314,621 SNPs (HumanHap300 bead chip, Illumina, San Diego, CA); and 2) 280 CAP and 652 PRINCE samples were genotyped for 620,901 SNPs (HumanQuad610 bead chip (Illumina). Both bead chips were designed to tag common genomic variation in Caucasians. Additional genotypes were obtained in 292 CAP and 634 PRINCE samples that were genotyped at 12,959 sites using a custom-made iSelect chip (N = 926). These measurements were used to infer the genotypes for approximately 2.5 million SNPs typed in the HapMap (phase II [31]) CEU parents using the genotype imputation software BIMBAM [32], [33].

### Quantile regression

Quantile regression was used to estimate the slope for the k^th^ lipoprotein, BMI, or height quantile versus the GRS [34], and bootstrap resampling to estimate their corresponding variances and covariances [35], [36]. One-thousand bootstrap samples were drawn for their estimation. The test for whether the slopes increased or decreased with the percentile of the dependent variable was based on the linear contrast of the slope at the 5^th^, 6^th^,…, 95^th^ quantiles of the phenotype. All analyses were performed using Stata (version 11, StataCorp, College Station, TX). In the text, the terms “increase” and “decrease” are used in the mathematical description of a function only, and do not imply actual phenotypic changes over time.

### Estimating the proportion of the variance explained

The classical regression model assumes the same regression slope applies to all quantiles of the independent variable. This means that when adjusting for the effect of the independent variable, either to control for its effect in multivariate analyses or to estimate the proportion of the variance it explains, it is unnecessary to specify a value of the independent variable to which the observations were adjusted. This is because the points maintain their same relative positions when they are projected to a common value along parallel trajectories, so all common values yield the same results. When the regression slopes are not parallel, the relative positions of the data points will change depending upon the value of the independent variable to which the points are projected. In the current analyses, the proportion of the variance explained by the GRS was computed by projecting all observations to the mean GRS value. Specifically, for each observation we: 1) determined the percentile rank of the lipoprotein values within its GRS decile; 2) determined the corresponding regression slopes; 3) determined the difference between the GRS and the mean GRS for the entire sample; and 4) subtracted their products from the original lipoprotein values. The regression slopes for noninteger quantile values were found by interpolation. The proportion of the variance explained by an individual SNP was computed the same way, except that the observed lipoprotein values were ranked within each genotype, and adjusted to the mean number of doses of the risk allele.
